# Creep and Shrinkage Behaviour of Disintegrated and Non-Disintegrated Cement Mortar

**DOI:** 10.3390/ma14247510

**Published:** 2021-12-07

**Authors:** Rihards Gailitis, Beata Figiela, Kalvis Abelkalns, Andina Sprince, Genadijs Sahmenko, Marta Choinska, Martin Duarte Guigou

**Affiliations:** 1 Faculty of Civil Engineering, Riga Technical University, Kalku 1, LV-1658 Riga, Latvia; kalvis.abelkalns@gmail.com (K.A.); andina.sprince@rtu.lv (A.S.); genadijs.sahmenko@rtu.lv (G.S.); 2 Faculty of Materials Engineering and Physics, Cracow University of Technology, Al. Jana Pawła II 37, 31-864 Kraków, Poland; 3 Research Institute in Civil and Mechanical Engineering GeM—UMR CNRS 6183, 58, Nantes University—IUT Saint-Nazaire, Rue Michel Ange, 44 600 Saint Nazaire, France; marta.choinska@univ-nantes.fr; 4 Department of Engineering and Technology, Catholic University of Uruguay, Av. 8 de Octubre 2738, Montevideo 11600, Uruguay; martin.duarte@ucu.edu.uy

**Keywords:** creep, shrinkage, long-term properties, disintegrated cement, non-disintegrated cement

## Abstract

One way to prevent cement from ending up in landfills after its shelf life is to regain its activity and reuse it as a binder. As has been discovered, milling by planetary ball mill is not effective. Grinding by collision is considered a more efficient way to refine brittle material and, in the case of cement, to regain its activity. There has been considerable research regarding the partial replacement of cement using disintegrated cement in mortar or concrete in the past few decades. This article determines and compares the creep and shrinkage properties of cement mortar specimens made from old disintegrated, old non-disintegrated, and new non-disintegrated Portland cement. The tests show that the creep strains for old disintegrated and old non-disintegrated cement mortars are close, within a 2% margin of each other. However, the creep strains for new non-disintegrated cement mortar are 30% lower. Shrinkage for old disintegrated and non-disintegrated cement mortar is 20% lower than for new non-disintegrated cement mortar. The research shows that disintegration is a viable procedure to make old cement suitable for structural application from a long-term property standpoint. Additionally, it increases cement mortar compressive strength by 49% if the cement is disintegrated together with sand.

## 1. Introduction

There has been a significant amount of research and industrial development in the material processing field to achieve the construction components’ longevity, reduce resource consumption and pollution caused by new material production, and reduce waste management costs [1]. These are significant issues in construction businesses because the ordinary Portland cement, considered the main binder material used in construction, is responsible for 5 to 8% of yearly global CO_2_ emissions. In 2020, it was expected that global cement production would reach 5.9 billion tons, and with that would come more than 4.8 billion tons of CO_2_ [2,3,4].

One way to refine and make used or old materials into fine powders is to use mills. Traditionally ball and planetary ball mills are used. However, these mills have weaknesses; for example, there is extensive wear of grinding parts of the mills, as the materials are exposed to intense heat, etc. Grinding by collision is considered a more effective way to refine brittle materials and an alternative for the mills that produce mineral supplements for construction mixtures. This method is referred to as disintegrator technology, where the material is milled by highly effective high-speed and high-intensity impact in contrast to low-speed impact abrasion [5]. Milling material this way also activates materials that, due to expired shelf life, have lost their ability to create a sufficient bonding effect [1]. One way to verify cement milling results and whether the old cement activity has been regained to the previous level is to assess setting characteristics. As the hydration temperature is closely tied to the cementitious material setting, it is considered one of the easiest methods to determine setting times and temperatures of cement-based materials. For this purpose, semi-adiabatic calorimetry (SAC) is used. SAC is a process that measures and records hydration specimen temperatures. Cement pastes, mortars, and concrete can be tested [6].

Creep and shrinkage are considered to be the main long-term properties that affect such human-made materials as concrete. Most of the creep and shrinkage develops in the first decade after construction. Afterward, developed strains are not expected to impact the structural performance significantly [7,8,9,10,11]. The creep in concrete structures can substantially influence the lifespan of the structure. It is commonly assumed to be a process that leads to increased deformations without significant damage development [9,10,11,12].

Many factors affect the nature of creep in concrete. The main factors are mixture proportions, curing age, environmental temperature, relative humidity, and applied stress level [7].

Shrinkage is a decrease in volume of cementitious material that occurs without external loads. Shrinkage and also drying shrinkage are factors that influence long-term strains. It has been assumed that in normal-strength concrete, the entire shrinkage strain is from drying shrinkage, and any contribution from autogenous shrinkage can be disregarded [13]. It has been determined that shrinkage strains are mainly caused by capillary pressure in the pore walls, according to capillary tension theory [14,15,16]. Concrete is restrained by tensile stress when the restraining body constrains shrinkage; thus, cracks appear when this restriction is exceeded. Because of this concrete mechanism occurring at an early age, shrinkage is considered one of the main reasons for crack development. It depends on the concrete’s aggregate type and content and the degree of hydration of cementitious material. The moisture movement is related to the restraining action of the aggregate particles [17,18,19].

Various testing codes and recommendations that are available for creep and shrinkage tests, such as RILEM TC 107, ASTM C512/C512M-10, and ASTM C157/C157M, state the conditions that have to be fulfilled for creep and shrinkage tests [20,21,22]. Additionally, the level of the load must be determined for the test application. Research shows that the theory of linear visco-elasticity is sufficient for creep deformation modelling. The condition here is that the stresses in concrete have to be limited to a level not higher than 40% of the concrete’s compressive strength, as required in Eurocode 2 [9].

This research aims to continue research in the field of disintegration product application carried out by Bumanis G., Dvorak K., and others [23,24,25,26] and to fill the gap in long-term property assessment with materials made from disintegration products. Furthermore, the authors aim to determine whether disintegration is a valid procedure that can return activity to old cement and whether cement that has been disintegrated shows any improvement in contrast to old cement. Additionally, the article aims to determine whether disintegration improves cement’s long-term properties.

## 2. Materials and Methods

**Figure 1:**
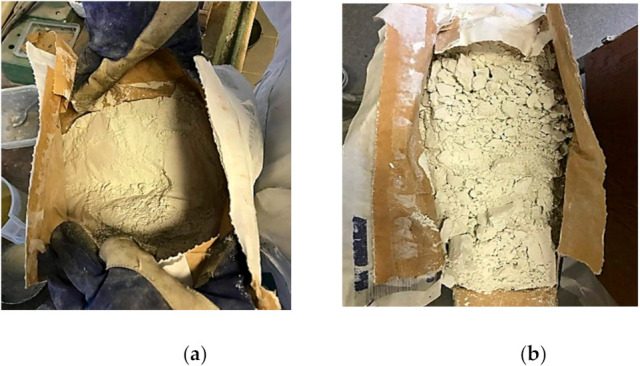
Fresh and old cement: (**a**) Fresh Aalborg white cement, (**b**) Ten-year-old Aalborg white cement.

For testing purposes, three cement composite mixes based on Aalborg white Portland cement were prepared. One group of specimens was new cement with sand that had not been subjected to grinding/activation with the disintegrator. Another was old cement with sand that had not been subjected to disintegration, and the last one was old cement with sand that had been subjected to disintegration. The old Aalborg cement had been stored in the laboratory for ten years. After those ten years, it had hydrated on the surface due to packaging and relative humidity changes in the room. The state of the new and old cement is shown in Figure 1.

**Figure 2:**
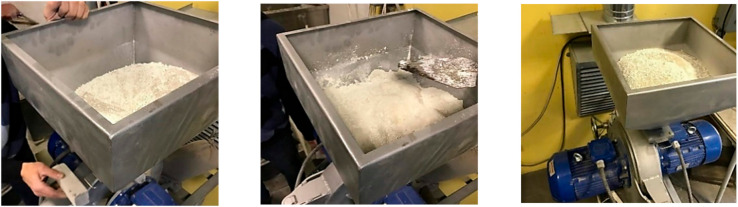
Disintegration process in DESI-16C disintegrator.

A disintegrated sand and cement mix was prepared in a DESI-16C disintegrator. Before the dry material was loaded into the disintegrator, the cement was sifted out through a sieve with 2 mm holes. Afterward, the cement and sand were mixed so that by the time they went through the disintegrator, they mixed even better, promoting finer milling of the cement grain. The linear impact speed in the disintegrator was 100 m/s. The disintegration process is shown in Figure 2.

**Figure 3:**
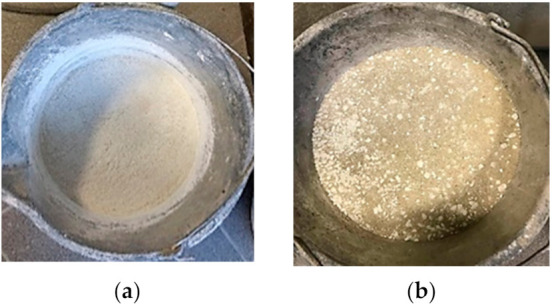
Disintegrated and non-disintegrated old cement with sand: (**a**) disintegrated old cement with sand; (**b**) non-disintegrated old cement with sand.

The mix differences between the disintegrated old cement with sand and non-disintegrated old cement with sand are visible in Figure 3.

After the disintegration process, the dry samples of each mix were XRD tested and mixes of disintegrated and non-disintegrated compounds were made.

The X-ray diffraction analyses were performed with the help of Rigaku Ultima + X-ray diffractometry equipment, which uses copper cathode radiation. The scanning speed of the sample was 4 degrees/min. The voltage was 40 kV, current −5 mA, 2θ scanning angle range from 5–60°. Data processing software (Software) Jade MDI 9 and electronic ICDD databases (PDF-4 + 2020, PDF-4/Organics 2020) were used to identify and qualitatively analyse the test substance, thus comparing the obtained data with the database file.

The mix proportions are shown in Table 1.

The mixes were poured into cylindrical moulds with dimensions Ø 46 × 250 mm according to RILEM TC 107. For testing purposes, more than thirty cylindrical moulds were prepared and filled. Specimens were unmoulded 24 h after they were poured.

### 2.1. Test Specimen Preparation

When the cylinders were unmoulded, they were Ø 46 × 250 mm as the moulds were. At 1 day after unmoulding, specimens were cut and ground according to RILEM TC 107 recommendation requirements for long-term testing in compression; this is a diameter to height ratio of 1/4 [20]. For this necessity, specimens were cut to the length of 194 mm approximately. Their top and bottom surfaces were ground, so that specimen height was 190 mm, and the top and bottom planes were perpendicular to the specimen’s longitudinal axes. Afterward, specimens were cured according to EN 12390-2 in water until day 6 when they were removed from the water.

**Figure 4:**
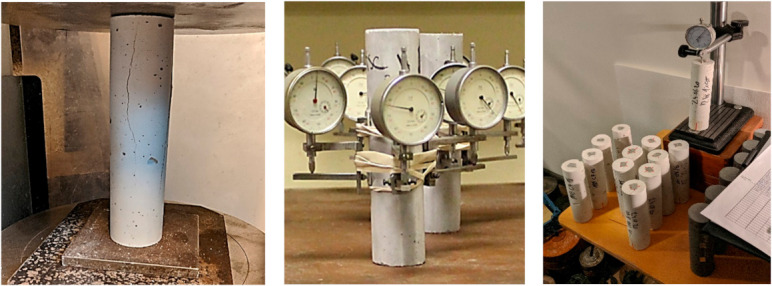
Compressive strength determination and prepared creep and shrinkage specimens [27].

When specimens reached 7 days of age, they were prepared for creep and shrinkage tests, and their compressive strength was determined. The compressive strength was determined using a compression machine with an accuracy of ± 0.01 MPa and loading rate of 0.7 MPa/s. The specimens meant for long-term tests had six aluminium plates (10 × 15 mm) glued on. The plates were glued so that deformation gauges had a proper grip on the specimens and could obtain correct readings. Prepared creep and shrinkage specimens are shown in Figure 4. Three specimens from each mix were tested for their compressive strength value, which is necessary to calculate the load that the specimens must be subjected to.

### 2.2. Experimental Setup

**Figure 5:**
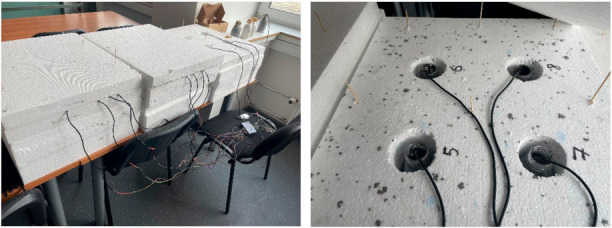
Semi-adiabatic calorimetry test setup.

Simultaneously with long-term specimen moulding, cases for semi-adiabatic calorimetry tests were also poured. This test was undertaken to verify that disintegration does activate cement that has been stored beyond its shelf life. In each container, 0.33 L of each mix was poured. The mix container holder was lined with insulation, and mix containers were placed inside. Afterward, the insulation material was also positioned above the containers. Temperature probes were placed in each of the mortar containers, and the hydration temperature was measured. Temperature readings were taken every 60 s, and the tests lasted for 40 h. The test setup is shown in Figure 5. Additionally, qualitative XRD analysis and PSA tests were undertaken to determine whether there were any crystalline phases in the cement and whether disintegration would decrease the particle size of the compounds.

**Figure 6:**
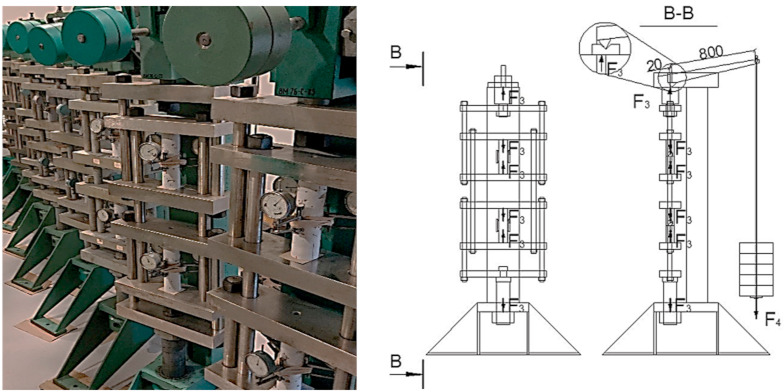
Creep specimen placement on the test stand.

The prepared cylindrical specimens were tested in creep lever test stands, shown in Figure 6.

Specimens in compression were loaded with 20% of the compressive strength value. All creep specimens were loaded in the linear strain state in the specimen-specific stress state [9].

Simultaneously with creep testing, shrinkage measurements were also carried out. The shrinkage specimens were placed alongside the creep specimens, but they were not subjected to load. The shrinkage strain readings were registered at the same time as the creep readings.

The creep and shrinkage tests were carried out in a controlled environment with a temperature of 21 ± 1 °C and relative humidity of 20 ± 3%.

## 3. Results

After the disintegration process for each mix, a PSA test was done to determine the particle size of the compositions.

**Figure 7:**
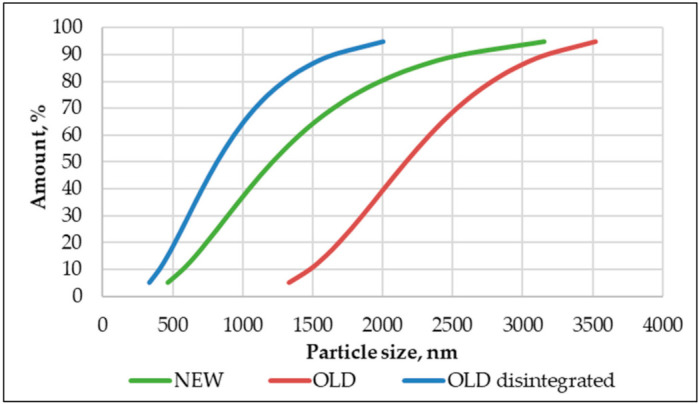
PSA analysis of non-disintegrated new, non-disintegrated old, and disintegrated old cement.

As is apparent in Figure 7, disintegration reduced particle size by almost 2 times. Additionally, the scattering of particle sizes in the old non-disintegrated mix was significantly larger than for the old disintegrated mix. The particle size for the old non-disintegrated mix was scattered from 1300 nm up to 3500 nm, whereas the old disintegrated cement particle size was scattered from 300 nm up to 2000 nm. It also must be acknowledged that disintegration made cement finer than the cement from the bag. One significant benefit of disintegration became apparent when specimens were moulded. The disintegrated mix was finer, and the moulding was more accessible than for non-disintegrated old (particle size: 1300 nm–3550 nm) and new (particle size: 450 nm–3200 nm) mixes. The grain size significantly improved the formability of the mixture.

**Figure 8:**
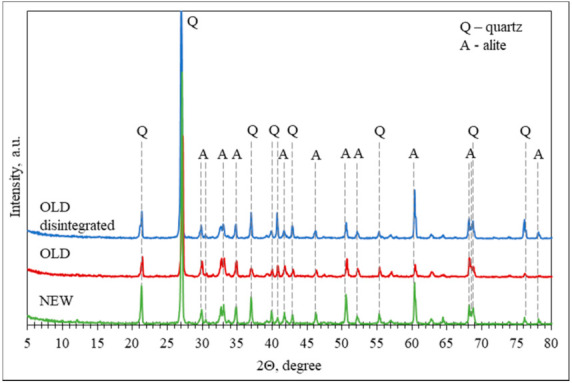
XRD patterns of studied cement composite dry compositions.

X-ray diffractograms of old disintegrated, old non-disintegrated, and new non-disintegrated cement and quartz sand mixtures (solid raw mixtures from Table 1) are presented in Figure 8. In all three cases, the presence of amorphous compounds (C-S-H and/or C-A-S-H), was not detected. Hence it can be argued that there were no visible signs of cement hydration products in all three cement composite solid raw compositions.

As the XRD was performed for solid raw compositions, relatively high intensity peaks of crystalline SiO_2_, also known as quartz, were detected. Additionally, all three compositions presented a presence of the crystalline phase of alite (the major phase in Portland cement that is responsible for early strength). After disintegration the intensity of alite increased. This could be explained by an increase in the specific surface area and activity of the cement mineral as a result of the disintegration process.

PSA and XRD tests for the tested sample dry mixes were undertaken within 12 h after cement composite mix disintegration.

**Figure 9:**
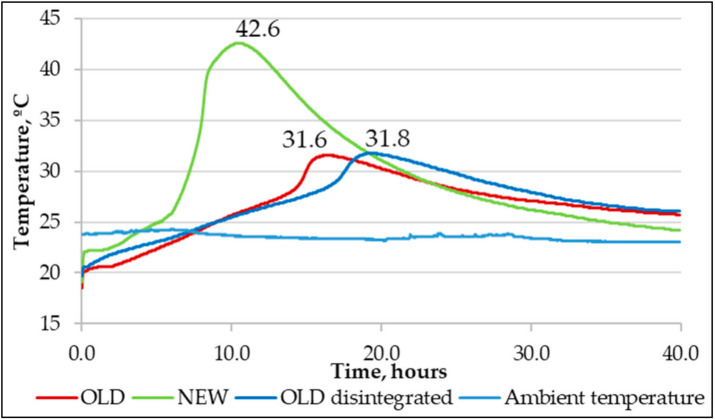
Non-disintegrated and disintegrated cement and sand hydration temperatures.

At the beginning of testing, a semi-adiabatic calorimetry test was undertaken. As a result, Figure 9 was acquired.

The curves show that the disintegration effect on hydration temperature development was not significant, and it seems that the activation effect was not noticeable. In contrast to the new cement, the old one had a 25% lower hydration temperature, which means that the old cement was 25% less active. The disintegrated mix had less than a 1% difference in hydration temperature from a non-disintegrated mix. That leads to thinking that one disintegration cycle did not significantly activate the old cement. Before the creep and shrinkage tests, compressive strength values were determined. The compressive strength test was carried out for specimens at the age of 7 days. Specimens were loaded at a rate of 0.6 ± 0.2 MPa/s. The results of compressive strength tests are compiled in Table 2.

**Figure 10:**
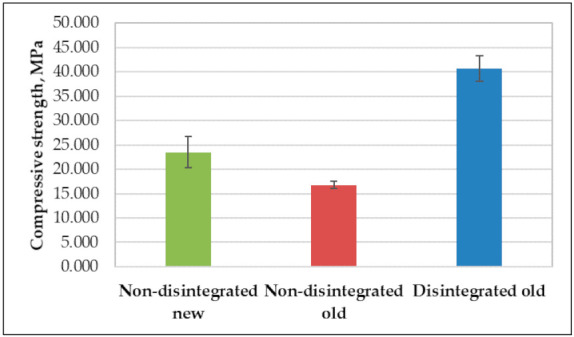
Average compressive strength values of tested specimens.

As is apparent from the compressive strength values in Table 2 and Figure 10, the disintegrated cement mortar had a higher compressive strength value. However, the calorimetry test did not show significant superiority over non-disintegrated old cement mortar. Here the disintegrated old cement mortar showed a compressive strength that was 49% higher than that of non-disintegrated old cement mortar and 31% higher than that of non-disintegrated new cement mortar. As for the compressive strength uncertainty for compositions, these were 8.4%, 4.3%, and 6.5% for non-disintegrated new, non-disintegrated old, and disintegrated old cement mortars, correspondingly. Additionally, it was quite noticeable that disintegrated old cement mortar specimens were by far the heaviest of the tested specimens. They were, on average, 12.4% and 24.7% heavier than old non-disintegrated cement mortar and new non-disintegrated cement mortar specimens, correspondingly. The disintegrator significantly impacted the compound’s granulometry change when cement and sand were disintegrated together. The finer grain of the old disintegrated cement and sand (400 nm–2000 nm) affected the compressive strength, which was 44% higher than for the new non-disintegrated cement with a particle size of 450 nm–3200 nm. This means that the disintegration of cement and sand together refined the sand and cement and made a compound with self-compacting abilities that was less likely to trap air in its structure and made a stronger mortar at the end.

Compressive strength results on day 7 (Figure 10) corresponded to the results of XRD (Figure 8). The higher compressive strength presented by the cement composite prepared using disintegrated old cement can be explained by the most intense diffraction peaks presenting the presence of alite (Figure 8).

According to these strength values, the creep specimens were loaded with 20% compressive strength for testing purposes. The long-term tests were carried out for 62 days.

**Figure 11:**
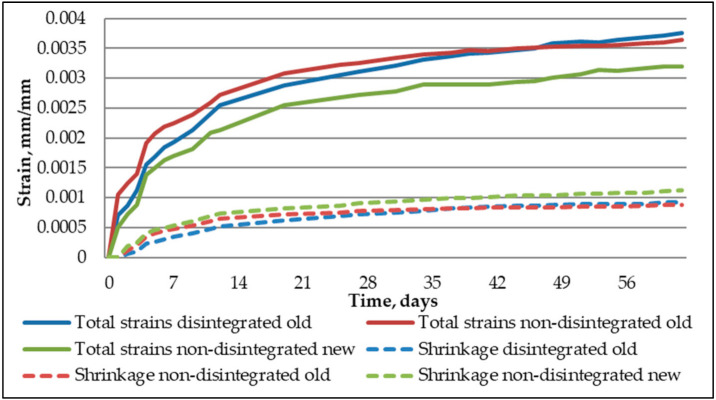
Total strains and shrinkage strains of tested cement composites.

Figure 11 shows the strain readings from shrinkage specimens and creep specimens.

**Figure 12:**
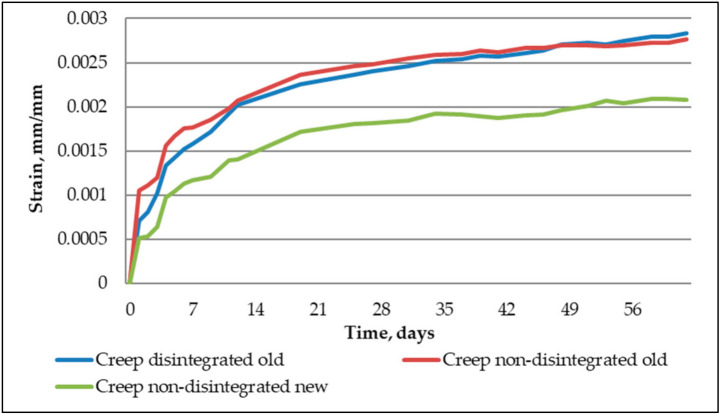
Creep strains of tested cement composites.

As is visible, the shrinkage strains were significantly smaller than the total strains from loaded specimens. The total strain readings contain shrinkage as well. This way, the results are more reliable if creep specimens were not wrapped in water evaporation restraining materials or coated with some other solution that could interact and change the compression’s raw material properties. As the shrinkage specimens were kept in the same environment, it is a matter of taking out of the full strain shrinkage strain, and the raw creep strains are obtained, as shown in Figure 12.

Additionally, it becomes apparent that shrinkage specimens did not achieve their inner humidity balance with the relative humidity in the laboratory. The strains kept on rising throughout the testing time.

From Figure 12, it is determined that creep strains to disintegrated and non-disintegrated cement mortars were quite close. As for non-disintegrated new cement, mortar creep strains at their peak were 26% lower than for disintegrated and non-disintegrated cement mortars. This characteristic means that to correctly evaluate the disintegration role in creep strain development, it was necessary to assess all the specimens’ specific creep.

Specific creep is calculated by dividing strains with applied stress:

(1) $$\chi_{cr}\left(t,t_{0}\right)=\frac{\varepsilon_{cr}\left(t,t_{0}\right)}{\sigma }=\frac{\varepsilon_{kop}\left(t\right)-\varepsilon_{sh}\left(t\right)-\varepsilon_{el}\left(t,t_{0}\right)}{\sigma }=\frac{1}{E_{cr}\left(t,t_{0}\right)}$$

$\chi_{cr}\left(t,t_{0}\right)$ is a specific creep,

$\varepsilon_{cr}\left(t,t_{0}\right)$ is a creep strain,

$\varepsilon_{kop}\left(t\right)$ is a total strain,

$\varepsilon_{sh}\left(t\right)$ is shrinkage strain,

$\varepsilon_{el}\left(t,t_{0}\right)$ is an elastic strain,

σ is the compressive stress,

$E_{cr}\left(t,t_{0}\right)$ is the modulus of creep.

**Figure 13:**
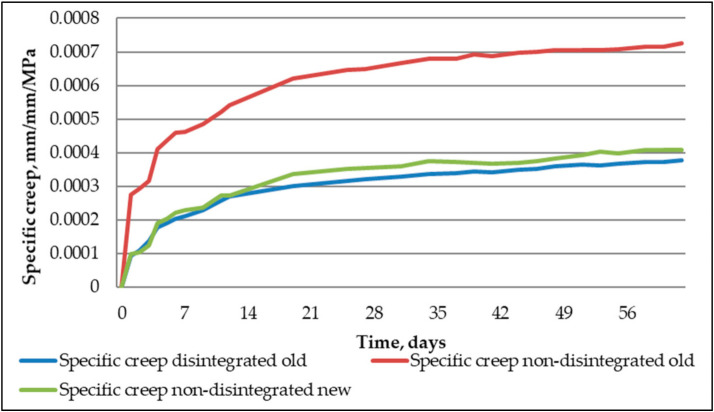
Specific creep of tested mortar specimens.

The actual creep properties of the three tested mortars show when the stress influence is taken out from the strains. In Figure 13, the specific creep of new non-disintegrated and old disintegrated cement mortar is very close. The difference at the peak is 7% in favour of disintegrated old cement mortar. As for the non-disintegrated old cement mortar, the expired shelf life of the cement shows clearly. The difference between non-disintegrated old and new cement mortars is 44%, and the difference between disintegrated and non-disintegrated old cement mortar is even higher—51%. The research shows that disintegration is a viable procedure for making old cement suitable for structural application from a long-term property standpoint.

## 4. Discussion

The currently conducted research on creep and shrinkage of concrete materials concerns the use of re-activated old cement to produce concrete mortars. Table 3 presents a comparison of the research results analysed from similar literature on the subject.

The results show the possibility of using disintegration as a process to improve raw material properties and regain properties of old materials to be used as raw materials once again. Additionally, the use of concrete aggregates from construction and demolition waste to produce new cement mortars correlates with this claim. Previous research has determined that the disintegration of sand does increase material activity and improves mortar compressive strength. Additionally, it has been noticed that partial replacement with recycled aggregate and the addition of shrinkage-reducing admixtures have a positive effect on the mechanical properties and change the tendency of creep kinetics [31]. In addition, the use of these materials contributes to the protection of the environment and supports the circular economy.

An interesting future development of the research carried out in this article would be the use and property assessment of recycled concrete aggregates and old disintegrated cement in building materials or elements of transport infrastructure. Combining these two methods would bring measurable economic and environmental benefits.

## 5. Conclusions

The main purpose of the research is to determine the disintegration influence of recovering cement properties. It also aims to evaluate the differences in long-term properties between old cement, new cement, and old cement that has been disintegrated:

- Disintegration does not show activity regeneration in old cement. There is a less than 1% difference in the hydration temperatures of non-disintegrated old and disintegrated old cement mortars and these are 25.4% lower than that of non-disintegrated new cement mortar. This further confirms that one cycle of collision milling does not affect the chemical composition of old cement.
- Disintegrated old cement mortar shows a 31% higher compressive strength than non-disintegrated new cement mortar whereas non-disintegrated old cement mortar shows an 18% lower compressive strength than non-disintegrated new cement mortar.
- Shrinkage strain readings show 21.8% and 17.5% larger strains to non-disintegrated new cement mortar and old non-disintegrated cement mortar than disintegrated old cement mortar. It means that shrinkage can be reduced by having finer base material and therefore better packing in the mould. The hydration temperature in this case does not have a significant role.
- New non-disintegrated cement mortar shows a vastly lower amount of creep strain than disintegrated old and non-disintegrated cement mortar; 26% and 26.1% less, correspondingly. However, when the stress impact to strains is taken out, or, in other words, specific creep is calculated, the most significant reduction in specific creep value is to that of old disintegrated cement mortar, with 7% and 51% increases in specific creep in new non-disintegrated and old non-disintegrated cement mortars, respectively. This means that disintegration makes old cement mortar 51% less willing to creep than non-disintegrated cement mortar and 7% less willing to creep than new cement mortar.
- Together with XRD analysis, it is safe to conclude from our study that proper storage of cement does not significantly reduce its properties over a short period. The disintegration effect on material properties is more related to obtaining finer material with a higher specific surface area after milling than it had before disintegration. Applying disintegration to old cement does not regain its chemical activity but it does make cement significantly finer and therefore ensures better packing of the compound into moulds, which decreases porosity and other heterogeneities of elements.

## Figures and Tables

**Table 1:** Prepared mix compositions.

Ingredients	Units	New Non-Disintegrated Cement Composite	Old Non-Disintegrated Cement Composite	Old Disintegrated Cement Composite
		Mass Proportion	Mass Proportion	Mass Proportion
New cement CEM I 52.5R	kg/m^3^	1.0	-	-
Old cement CEM I 52.5R	kg/m^3^	-	1.0	-
Old disintegrated cement CEM I 52.5R	kg/m^3^	-	-	1.0
Quartz sand 0.4/1 mm	kg/m^3^	1.75	1.75	-
Quartz sand 0/0.5 mm	kg/m^3^	1.2	1.2	-
Disintegrated quartz sand 0.4/1 mm	kg/m^3^	-	-	1.75
Disintegrated quartz sand 0/0.5 mm	kg/m^3^	-	-	1.2
Water	kg/m^3^	0.5	0.5	0.5
Plasticizer Stachema	kg/m^3^	0.004	0.004	0.004
W/C ratio C/S	- -	1/2 1/3	1/2 1/3	1/2 1/3

**Table 2:** Ultimate load values for all tested specimen types.

Mix Type	Average Mass, kg	Average Compressive Strength on Day 7, MPa	Average Density at the Age of 7 Days, kg/m^3^
Non-disintegrated old	0.3283	19.07	2136.95
Disintegrated old	0.3749	37.42	2453.43
Non-disintegrated new	0.2824	25.83	2171.79

**Table 3:** Summary of literature results on concrete creep and shrinkage tests.

Materials	Results			Source
	Creep Strains	Shrinkage Strains	Compressive Strength [MPa]	
Non-disintegrated new cement mortar with sand	New cement mortar specimens showed vastly better creep properties, with 26 and 26.1% less than disintegrated and non-disintegrated old cement mortars.	The highest shrinkage occurred with new cement mortar, followed by old non-disintegrated and old disintegrated cement mortars correspondingly by 21.8 and 17.5% less.	Disintegrated cement mortar showed superior compressive strength over new cement and old cement non-disintegrated cement mortars by 31 and 49%, respectively.	
Non-disintegrated old cement mortar with sand				
Disintegrated old cement mortar with sand				
Cement, sand, water, natural aggregate concrete	Increasing the exchange of recycled coarse aggregate increased the basal and total creep deformation. The creep factor of old recycled aggregate concrete grew very quickly.	-	The best compressive strength was obtained for the sample with natural aggregate—40.2 MPa. Composites with recycled coarse aggregate achieved a compressive strength of 29 MPa, i.e., 27% less than the highest result.	[28]
Cement, sand, water, recycled coarse aggregate				
Portland cement, sand, fly ash, crushed granite, recycled aggregate	Concrete creep increased with increasing recycled aggregate content. The use of fly ash as a partial replacement reduced the creep kinetics of the concrete.	With the increase in the content of recycled aggregate, the shrinkage of concrete drying increased. The use of fly ash reduced shrinkage on drying in a blend with recycled aggregate to some extent.	The lowest strength was obtained for concrete with recycled coarse aggregate—19.4 MPa. On the other hand, the addition of crushed granite significantly increased the strength, by 43%. Thus, the recycled material can successfully fill in cement composites.	[29]
Portland cement, fly ash, sand, recycled aggregate				
Cement, natural coarse aggregate, fine aggregate	An increase in the replacement of recycled aggregate resulted in an increase in both creep and shrinkage of the material. It was related to the adhesion of the mortar.	The development of the shrinkage kinetic of mortar partially made of recycled grout increased by 25% and 48%, respectively, for the 50% and 100% substitute.	The addition of recycled coarse aggregate slightly reduced the compressive strength of the cement composite (by 7%), compared with concrete containing natural aggregate.	[30]
Cement, recycled coarse aggregate, fine aggregate				

## Data Availability

Data sharing is not applicable.
